# Colorectal Cancer screening in ambulatory healthcare service clinics in Abu Dhabi, United Arab Emirates in 2015–2016

**DOI:** 10.1186/s12885-021-08623-9

**Published:** 2021-08-06

**Authors:** Aysha Almansoori, Mariam Alzaabi, Latifa Alketbi

**Affiliations:** Ambulatory Healthcare Services, SEHA, Abu Dhabi, United Arab Emirates

**Keywords:** Colorectal cancer screening, Fecal immunochemical test (FIT), Guaiac fecal occult blood testing (gFOBT), Colonic polyps, Colorectal cancer

## Abstract

**Background:**

Colorectal cancer (CRC) is a major public health issue due to high morbidity and mortality. Different screening programs were implemented to reduce its burden.

**Objectives:**

To estimate the prevalence of CRC screening uptake using fecal immunochemical test (FIT) or guaiac fecal occult blood testing (gFOBT) in Emirati nationals. Other objectives were to measure the incidence of CRC in the screened population, to measure the outcomes of follow-up screening colonoscopy after positive FIT/gFOBT and to identify the causes of not performing follow-up screening colonoscopy after positive FIT/gFOBT.

**Methodology:**

Adult Emirati nationals aged 40–75 years who visited Ambulatory healthcare services clinics, Abu Dhabi in 2015–2016 were included in the study. The electronic medical records of the eligible individuals were reviewed retrospectively. The prevalence of CRC screening was measured among the eligible population using the FIT/gFOBT. The IBM SPSS Statistics program, version 21.0.0, was used for analysis.

**Result:**

45,147 unique individuals were eligible for screening, and only 23.5% were screened using FIT/gFOBT. Of the screened individuals, 13.5% had positive FIT/ gFOBT, and 30.5% of those underwent follow-up screening colonoscopy. CRC was diagnosed in 11 individuals. Colonic polyp were found in 30.5% of individuals who had undergone a follow-up colonoscopy. Collectively 933 individuals did not undergo follow-up screening colonoscopy after having a positive FIT/gFOBT, and about 36.3% had collected the result and referred to a gastroenterologist but did not attend the appointment.

**Conclusion:**

CRC screening uptake using FIT/gFOBT is low among the adult Emirati nationals.

## Background

Cancer is a major health issue in many countries worldwide, and it is the second leading cause of death worldwide [1]. Colorectal cancer (CRC) is the third most common malignancy and the fourth leading cause of cancer-related deaths worldwide [2]. The American Cancer Society data released in 2014 indicated that the incidence of colon cancer decreased by 30% among adults aged ≥50 years in the United States of America in the last 10 years owing to the increase in the frequency of performing colonoscopy [3]. The United States of America preventive task force (USPSTF) recommends CRC screening for adults aged 50 to 75 years by performing high-sensitivity guaiac fecal occult blood testing (gFOBT) or fecal immunochemical test (FIT) every year, sigmoidoscopy with high-sensitivity gFOBT or FIT every 5 years, or colonoscopy every 10 years [4]. Multiple studies had shown that different CRC screening methods reduced colorectal cancer-associated mortality [5, 6].

In the United Arab Emirates (UAE), significant changes have occurred over the last 40 years in different sectors including the economic, social, and demographic sectors. These changes led to improvement in the healthcare sector, thereby resulting in an increase in the life expectancy, decrease in the incidence of communicable diseases, and increase in the incidence of non-communicable diseases including cancer, which became the third leading cause of death in the UAE in 2010 [7]. The incidence of cancer in the UAE is expected to increase owing to aging and increase in the exposure to risk factors of cancer [7]. The incidence of CRC had been increasing in the Emirate of Abu Dhabi, from 157 cases in 2012 (25% of cases are Emirati patients), 173 cases in 2013 (24% of cases are Emirati patients), to 175 cases in 2015, making it the third most common cancer in both sexes; CRC was the most common cancer in men (117 cases) and the third most common in women (58 cases) in the Emirate of Abu Dhabi [8–10]. In 2016, colon cancer caused 6.9% of all cancer-related deaths (7.7% in men and 6% in women) in the Emirate of Abu Dhabi [10].

The current study aimed to estimate the prevalence of CRC screening uptake using FIT or gFOBT in eligible adult Emirati population (aged 40–75 years) who visited ambulatory healthcare clinics in the Emirate of Abu Dhabi between September 2015 and September 2016. The secondary aims were to determine the incidence of CRC in the screened population (aged 40–75 years), to measure the outcomes of follow-up screening colonoscopy after positive results on FIT/gFOBT, and to measure the prevalence and histology of colonic polyps diagnosed on follow-up screening colonoscopy; and to identify the causes of not performing follow-up screening colonoscopy after positive results on FIT/gFOBT.

## Methods

In 2013, the Health Authority - Abu Dhabi (HAAD currently Known as Department of Health Abu Dhabi) launched the CRC screening program for UAE nationals aged 40–75 years to reduce the morbidity and mortality due to CRC [11]. According to the HAAD standard for CRC screening, individuals at average risk of CRC (i.e., those aged > 40 years, with no history of adenoma or CRC, no history of inflammatory bowel disease, and negative family history for CRC) are recommended to undergo screening colonoscopy every 10 years or FIT every 2 years if the patient refuses to undergo screening colonoscopy. Individuals should be screened with colonoscopy and other investigations should be performed according to their medical conditions if they are at an increased risk of CRC (i.e., those with a history of adenoma, sessile serrated polyps, inflammatory bowel disease, family history of first- or second-degree relatives with CRC or history of CRC) or at high risk of CRC (i.e., those with hereditary non-polyposis CRC and those with polyposis syndromes such as classical familial adenomatous polyposis, familial adenomatous polyposis, *MYH*-associated polyposis, PeutzJeghers syndrome, juvenile polyposis syndrome, and hyperplastic polyposis syndrome). According to HAAD CRC standards, the percentage of Abu Dhabi population who should undergo screening should be 45%, with a preferred percentage of 65% of the eligible population. The maximum time between referral after a positive result on screening FIT and the follow-up colonoscopy should be 31 days in > 90% of the screened individuals according to the standard [11]. As per the new Department of Health (DOH) CRC screening program specifications in 2019, individuals at average risk of CRC are to be screened by FIT annually if they refused to undergo screening colonoscopy [12]. As Per the HAAD statistics, the total population in Abu Dhabi was estimated to be 2,486,402 (551,535 Emirati nationals) in year 2016, with the total of 92,380 Emirati nationals aged 40–74 years in the same year [10].

SEHA is the main governmental healthcare provider in the Emirate of Abu Dhabi. Ambulatory health service (AHS) is the main primary healthcare provider under SEHA company, which operates more than 24 ambulatory and primary healthcare clinics, across the whole Emirate of Abu Dhabi. Each individual is identified across all SEHA facilities by using a unique identifier—i.e., the enterprise patient identifier (EPI)—that can link different medical records for each individual [13].

Adult Emirati nationals aged 40–75 years (both ages included) who visited AHS clinics under SEHA in the Emirate of Abu Dhabi between September 1, 2015 and September 30, 2016 were included in the study. Non-nationals, those aged < 40 years or > 75 years, and those who visited AHS clinics during any other time apart from the study period or who visited other medical centers were excluded.

The electronic medical records of the eligible individuals were reviewed retrospectively, and the required data were extracted. Report was generated with multiple fields including the EPI, medical record number, age, body mass index (BMI), dates of FIT/ gFOBT and colonoscopy, presence of comorbidities, family history, and aspirin use. Individuals were considered to have been screened if they had undergone FIT/ gFOBT within 2 years, as recommended by the HAAD guidelines. Multiple encounters or clinic visits for the same individual were noted, with a total of 173,489 clinic encounters. The data were stratified considering the individuals’ encounters by EPI, keeping one encounter per individual. FIT was performed in majority of the patients, but if FIT was not available, gFOBT was performed. If the individual had a positive result on FIT/ gFOBT within 2 years of the study period, he/she was considered to have positive FIT/ gFOBT result. If two FIT/ gFOBT results were obtained and showed the same outcomes, the result obtained on the most recent date was considered. The final number of unique eligible individuals was 45,147, after excluding 128,342 duplications. The final report was coded in the AHS research office, and all individual identifications were removed. The de-identified data were then coded and analyzed in the AHS research office.

Information about bowel preparation for follow-up screening colonoscopy was obtained from the colonoscopy reports documented by the endoscopists. If bowel preparation was documented to be satisfactory, the colonoscopy results were considered. Colonoscopy results were not considered if the endoscopist reported poor bowel preparation, if the colonoscopy was not completed owing to patient intolerance or technical difficulties, or if colonoscopy results were recorded as indeterminate. If two or more colonoscopies were performed to screen for CRC after positive results on FIT/gFOBT, the first complete colonoscopy result was considered. All the colonoscopy findings were documented from colonoscopy reports, and the histology of the polyps was obtained from the histopathology reports.

If follow-up screening colonoscopy was not performed after a positive result of FIT/gFOBT, the individual electronic medical record was reviewed to determine the cause of not having a follow-up screening colonoscopy based on the documentation in the individual medical record. A unified classification was used to identify the possible factors of not having a follow-up colonoscopy after having a positive FIT/gFOBT. Some of the causes for not performing follow-up colonoscopy in individuals were as follows: the individual did not collect the result; the individual received the result but was not referred to a gastroenterologist and the reason was not documented in the medical records; the individual was referred to a gastroenterologist but missed the appointment; the individual was counseled by a gastroenterologist but was not advised to undergo a screening colonoscopy owing to different indications; the individual refused to undergo screening colonoscopy before or after being referred to a gastroenterologist; the individual had an appointment for a colonoscopy but missed the colonoscopy appointment; the individual had a medical condition and was being followed-up by the gastroenterologist who recommended no further investigation needed or the individual was known to have CRC.

IBM SPSS Statistics program, version 21.0.0, was used for statistical analysis Frequencies and cross tabulation were used with mean, median, interquartile range (IQR) and standard deviation (SD) being calculated for the continuous variables. In addition logistic regression analysis was used to study the binary outcomes. Association was considered significance at *p* value of less than 0.05. The current study was approved by the research committee of the AHS.

## Results

**Fig. 1:**
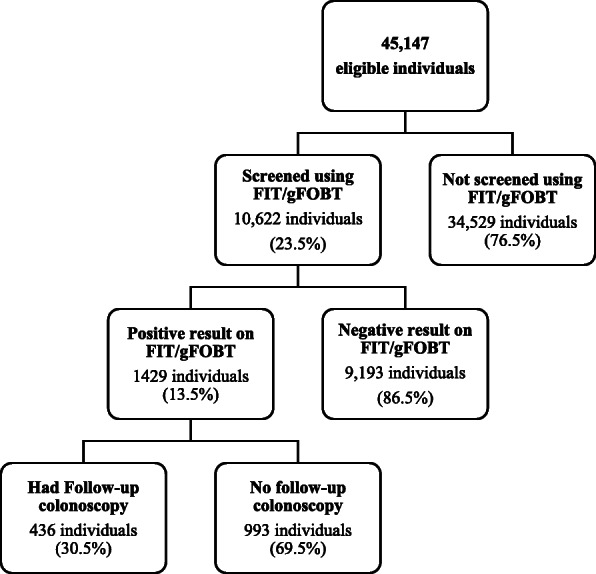
Flowchart of colorectal cancer screening eligible individuals using FIT/gFOBT outcome

A total of 45,147 unique individuals were eligible for screening (62% women and 38% men), with a mean age of 51.51 ± 9.3 years. Totally, 54.1% of the eligible individuals were less than 50 years of age (Table 1). During the study period, 23.5% of the eligible individuals were screened using FIT/gFOBT. Of the screened population, 13.5% had positive FIT/ gFOBT (Fig. 1). A total of 69.6% of the individuals with positive FIT/FOB were women. In addition, 3.55% of all the screened women and 25.33% of all screened men had positive results on FIT/ gFOBT. The mean age of patients who had positive FIT/ gFOBT was 56.48 ± 8.95 years (69.33 ± 6.43 years for men and 58 ± 11.29 years for women).

**Table 1:** Characteristics of eligible individuals

		Age_Groups				
	Character	< 50 years	51–59 years	60–69 years	70 years and older	Total
		n (%)	n (%)	n (%)	n (%)	n (%)
**FIT/gFOBT**	**Sex**					
**FIT /gFOBT not done**	Female	12,706 (36.8%)	4781 (13.8%)	2549 (7.4%)	587 (1.7%)	20,623 (59.7%)
	Male	8018 (23.2%)	3189 (9.2%)	2150 (6.2%)	544 (1.6%)	13,901 (40.3%)
**Negative result on FIT /gFOBT**	Female	2377 (25.9%)	2348 (25.5%)	1374 (14.9%)	252 (2.7%)	6351 (69.1%)
	Male	883 (9.6%)	882 (9.6%)	849 (9.2%)	228 (2.5%)	2842 (30.9%)
**Positive result on FIT /gFOBT**	Female	302 (21.1%)	358 (25.1%)	280 (19.6%)	54 (3.8%)	994 (69.60%)
	Male	120 (8.4%)	131 (9.2%)	145 (10.1%)	39 (2.7%)	435 (30.4%)
	Total	24,406 (54.1%)	11,689 (25.9%)	7347 (16.3%)	1704 (3.8%)	45,146 (100%)
	**Aspirin**					
**FIT /gFOBT not done**	No	19,062 (55.2%)	6067 (17.6%)	2633 (7.6%)	554 (1.6%)	28,316 (82%)
	YES	1662 (4.8%)	1903 (5.5%)	2066 (6%)	577 (1.7%)	6208 (18%)
**Negative result on FIT /gFOBT**	No	2912 (31.7%)	2240 (24.4%)	1059 (11.5%)	189 (2.1%)	6400 (69.6%)
	YES	348 (3.8%)	990 (10.8%)	1164 (12.7%)	291 (3.2%)	2793 (30.4%)
**Positive result on FIT /gFOBT**	No	371 (26%)	328 (23%)	201 (14.1%)	26 (1.8%)	926 (64.8%)
	YES	51 (3.6%)	161 (11.3%)	224 (15.7%)	67 (4.7%)	503 (35.2%)
	Total	24,406 (54.1%)	11,689 (25.9%)	7347 (16.3%)	1704 (3.8%)	45,146 (100%)
	**Family history of CRC**					
**FIT /gFOBT not done**	Negative	20,647 (59.8%)	7941 (23%)	4687 (13.6%)	1130 (3.3%)	34,405 (99.7%)
	Positive	77 (0.2%)	29 (0.1%)	12 (0.0003%)	1 (0.00002%)	119 (0.3%)
**Negative result on FIT /gFOBT**	Negative	3220 (35%)	3204 (34.9%)	2216 (24.1%)	479 (5.2%)	9119 (99.2%)
	Positive	40 (0.4%)	26 (0.3%)	7 (0.1%)	1 (0.0001%)	74 (0.8%)
**Positive result on FIT /gFOBT**	Negative	417 (29.2%)	483 (33.8%)	422 (29.5%)	92 (6.4%)	1414 (99%)
	Positive	5 (0.3%)	6 (0.4%)	3 (0.2%)	1 (0.1%)	15 (1%)
	Total	24,406 (54.1%)	11,689 (25.9%)	7347 (16.3%)	1704 (3.8%)	45,146 (100%)

**Fig. 2:**
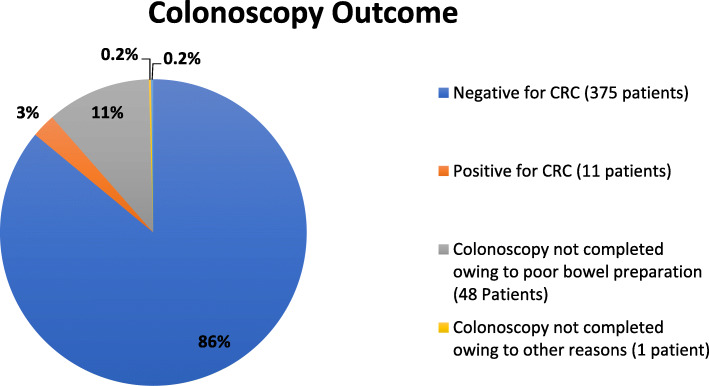
Outcomes of follow-up screening colonoscopy

A total of 436 individuals who had positive FIT/ gFOBT underwent follow-up screening colonoscopy. Totally, 11 individuals were diagnosed with colorectal cancer. Forty-nine individuals had incomplete colonoscopy, and one individual had indeterminate results on colonoscopy (Fig. 2).

**Fig. 3:**
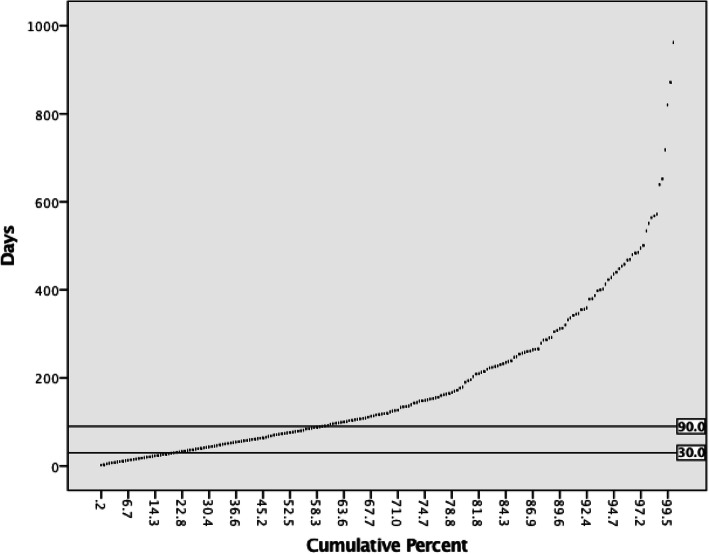
Interval (days) from showing a positive result on FIT/gFOBT to undergoing follow-up colonoscopy

The median time from showing a positive FIT/ gFOBT to the follow-up screening colonoscopy for patients who had positive FIT/gFOBT in days was 72 (interquartile range 114.25 days) (Table 2), More than 50% of individuals with a positive result on FIT/ gFOBT undergone follow-up screening colonoscopy within 90 days of obtaining the FIT/ gFOBT result (Fig. 3).

**Table 2:** Characteristics of individuals who showed positive FIT/ gFOBT and underwent follow-up screening colonoscopy

Descriptive		Age (years)		BMI (kg/m2)		Interval from positive FIT/ gFOBT to follow up screening colonoscopy (days)	
		Statistic	Std. Error	Statistic	Std. Error	Statistic	Std. Error
Mean		56.482	0.2366	31.2003	0.15704	125.5704	6.99963
95% Confidence Interval for Mean	Lower Bound	56.018		30.8923		111.8122	
	Upper Bound	56.946		31.5084		139.3286	
5% Trimmed Mean		56.432		30.9174		106.8928	
Median		56		30.48		72	
Variance		80.029		35.24		20,871.817	
Std. Deviation		8.9459		5.93633		144.47082	
Minimum		40		15.44		2	
Maximum		74		56.84		962	
Range		34		41.4		960	
Interquartile Range		15		7.08		114.25	
Skewness		0.044	0.065	0.794	0.065	2.34	0.118
Kurtosis		−1.027	0.129	1.079	0.129	6.658	0.236

Eleven individuals were diagnosed with CRC; 8 in women and 3 cases in men, with a mean age of 61.09 ± 11.2 years (range, 40–74 years). Two women were diagnosed with CRC at the ages of 40 and 41 years, respectively. The mean age of women diagnosed with CRC was 58 ± 11.29 years, while the mean age of men diagnosed with CRC was 69.33 ± 3 years. Age was found to be a risk factor for developing CRC statistically (odds ratio [OR], 1.082; *p* = 0.041), while sex, weight, family history, and aspirin use were not found as risk factors for CRC statistically (*p* = 0.75, 0.56, 0.55, and 0.31, respectively).

Different findings were reported on the follow-up screening colonoscopy reports including hemorrhoids (43.12%), colonic polyps (30.5%), diverticulosis (15.83%), colitis (3.44%), and colonic ulceration (3.21%). In addition, 17.2% had negative results on colonoscopy. Moreover, 4.82% of patients had gastritis or esophagitis and 0.23% had esophageal varices on esophagogastroduodenoscopy that was performed after negative colonoscopy to determine the possible causes for a positive FIT/ gFOBT (Table 3).

**Table 3:** Other colonoscopy and esophagogastroduodenoscopy findings (*n* = 436)

Other colonoscopy/esophagogastroduodenoscopy findings	No.	Percentage
Hemorrhoids	187	43.12%
Diverticulosis	68	15.83%
Polyps	133	30.50%
Colitis	15	3.44%
Colonic ulceration	14	3.21%
Gastritis or esophagitis	21	4.82%
Esophageal varices	1	0.23%
Others	9	2.06%
Negative finding	75	17.20%

Different colonic polyp types were diagnosed on histopathology, while 18 individuals had more than one type of polyps. Tubular adenoma with low-grade dysplasia was the most common type of polyp (58.65%). The other types included tubular adenoma with high-grade dysplasia (3.76%), tubulovillous adenoma with low-grade dysplasia (10.53%), tubulovillous adenoma with high-grade dysplasia (0.75%), hyperplastic polyps (23.31%), inflammatory polyps (1.50%), and others (15.04%; Table 4). Age and sex were identified as significant risk factors for developing CRC or colonic polyps (OR, 1.038; *p* = 0.002; and OR, 1.605; *p* = 0.034, respectively).

**Table 4:** Histology findings of colonic polyps (*n* = 133)

Colonic polyp Histology Diagnosis	No.	Percentage
Inflammatory polyp	2	1.50%
Hyperplastic polyp	31	23.31%
Tubular adenoma with low-grade dysplasia	78	58.65%
Tubular adenoma with focal high-grade dysplasia	5	3.76%
Tubulovillous adenoma with low-grade dysplasia	14	10.53%
Tubulovillous adenoma with high-grade dysplasia	1	0.75%
Others	20	15.04%

Approximately 69.5% of individuals who had positive FIT/gFOBT did not undergo a follow-up screening colonoscopy. A total of 12% of the individuals did not follow-up their result at the healthcare centers, 36.30% received the result and were referred to a gastroenterologist but did not attend the appointment with the gastroenterologist, 5.10% were known to have a gastrointestinal condition that could result in a positive FIT/ gFOBT or were followed-up by gastroenterologist who did not recommend a follow-up colonoscopy, and 11.3% refused to undergo follow-up screening colonoscopy (Table 5).

**Table 5:** Causes for not undergoing follow-up screening colonoscopy

Causes for not undergoing follow-up screening colonoscopy	Frequency	Percentage
Individual did not collect the result	119	12%
Individual was examined by the physician but the result was not collected	14	1.40%
Individual was referred to a gastroenterologist but did not visit the gastroenterologist	360	36.30%
Counselled by a gastroenterologist	104	10.50%
Refused colonoscopy	112	11.30%
Individual scheduled for colonoscopy, but did not undergo the procedure	92	9.30%
Individual followed-up by a gastroenterologist for a known gastrointestinal disease	51	5.10%
Others	136	13.70%
Known case of colorectal cancer	5	0.50%

## Discussion

A total of 45,147 individuals were eligible for CRC screening, accounting for 48.9% of the entire Emirati Abu Dhabi population of the same age group in 2016 (92,380) visited AHS centers in the study period. FIT/ gFOBT screening was performed in 23.5% of the eligible individuals, which was similar to the screening percentage (26.7%) observed in Singapore national survey that included 1763 individuals aged ≥50 years [14]. However, the screening percentage in our study was lower than that obtained in the 2011 UK Bowel screening program (UKBSP), which was 52% [15]. In the United States of America, the incidence of CRC screening (using FOBT within the past year or lower endoscopy [sigmoidoscopy or colonoscopy] within the last 10 years) for adults aged 50–75 years was 62.9% in 2008 [16]. The low rate obtained in the current study is alarming and may reflect the lack of knowledge in the UAE community about CRC screening and the need to implement effective measures to increase the CRC screening rate [17–19].

Totally, 30.5% of individuals (436 individuals) underwent follow-up screening colonoscopy after showing a positive FIT/gFOBT; this value is lower compared to 98.1% (17,192) of individuals who underwent a follow-up colonoscopy in the UKBSP study [15].

This low rate can be attributed to the patient’s failure to follow-up of the result, their lack of knowledge about CRC, or their embarrassment to undergo screening colonoscopy, especially if a male endoscopist was to perform colonoscopy for a female patient as noted in few of the female medical records which showed that they have requested a female endoscopist. A systematic review has shown that the female gender has less CRC screening rate, and this issue needs further analysis among the UAE community to increase CRC screening [20].

Eleven cases of CRC were diagnosed: 3 in men and 8 in women, with a mean age of 61.09 ± 11.2 years (range, 40–74 years). In Sheikh Khalifa Medical City (SKMC), Abu Dhabi, 103 Emirati nationals (57.9% men) were diagnosed with colorectal adenocarcinomas between 2000 and 2011; the mean patient age was 57 years [21]. Men had a higher incidence of CRC in our study, similar to the HAAD data that showed that men are diagnosed with CRC more often [9]. Similar data was also observed in the UKBSP study where a higher number of men were diagnosed with CRC than women were [15]. In the current study, age was a risk factor for developing CRC (OR, 1.082; *p* = 0.041).

The current study showed that CRC occurred at a younger age in women (40 and 41 years), with the mean age at the time of CRC diagnosis being 58 ± 11.29 years for women and 69.33 ± 3 years for men. This finding is similar to that obtained in different studies conducted within UAE and the Gulf countries [22–24]. These findings support the recommendation of HAAD to start screening at 40 years of age, earlier than that followed in other countries [11].

About one third of individuals who undergone screening colonoscopy were diagnosed with colonic polyps, with tubular adenoma with low-grade dysplasia being the most common finding (58.65%). Similar findings were reported in the screening series of the UK Bowel Cancer Screening Program (BCSP), in which tubular adenoma was the most common type of polyps (48–55%), while other types of polyps were tubulovillous adenoma (15–24%), villous polyps (1–6%), or highgrade dysplasia (5–14%) [25]. The prevalence of colonic polyps was higher in the current study than in a study of patients who underwent bariatric surgery in SKMC, Abu Dhabi (341 patients) and who underwent colonoscopy for screening or for different indications which was 7.6% [22]. In that study, a total of 77% of the polyps were found in individuals with higher body mass index (BMI) (BMI > 30 kg/m^2^) than in other individuals (BMI < 30 kg/m^2^). The incidence of CRC (60%) and hyperplastic polyps was higher in individuals with higher BMI, although there was no statistical significance [22]. In a single-center study in Abu Dhabi involving 616 patients who underwent screening colonoscopy, the prevalence of polyps was 27% (13% for adenoma and 33% for hyperplastic polyps), while 17 patients (2.76%) aged 38–70 years were diagnosed with CRC during 2014–2015 [26].

A low rate of follow-up of abnormal FIT/gFOBT has been noted in different studies and different factors were attributed [26–30]. In our study, most of the patients were referred to a gastroenterologist, but they were not examined or counseled by the gastroenterologist. This suggests the lack of knowledge about the importance of follow-up which can put the patient at risk of having advanced CRC and increased mortality [26–29]. Some of the patients’ appointments were missed or not scheduled, which can be due to a lack of communication between the patients and the healthcare system that was addressed in multiple studies [30, 31]. A total of 12% of the patients were not informed about the positive FIT/gFOBT, which delays the timely medical intervention, reflecting the essential role of the healthcare provider to ensure follow-up [30]. One of the observations was repeating the FIT/ gFOBT if the result was positive, which may reflect the lack of patients’ trust in the test [27].

The study had several limitations including the retrospective nature of the study. All the data were extracted from electronic medical records, so record bias might be an issue if the physician did not document the appropriate management for the individuals who showed a positive FIT/gFOBT. In addition, only the AHS facilities in Abu Dhabi were included, and only considered FIT/gFOBT as the screening method while excluding direct screening colonoscopy. Moreover, the exact incidence of CRC diagnosed via FIT/gFOBT could not be determined, as we were unable to obtain the data about all CRC cases in Abu Dhabi during the same study period for measuring the effectiveness of the screening program using FIT/gFOBT.

## Conclusions

Colorectal screening uptake is low among the Emirati population in the Emirate of Abu Dhabi. Different interventions to increase the rate of colorectal cancer screening are needed to reduce colorectal cancer-associated morbidity and mortality. Accordingly, we recommend more studies regarding CRC risk factors and patterns in our community. Moreover, we need to increase awareness about CRC screening in our community.

## Data Availability

All the data and material are kept in Ambulatory Healthcare Services research office. The data that support the findings of this study are available from Ambulatory Healthcare Services research office but restrictions apply to the availability of these data, which were used under license for the current study, and so are not publicly available. Data are however available from the authors upon reasonable request and with permission of Ambulatory Healthcare Services (AHS); Al Ain Medical District Human Research Ethics Committee, after the approval of the Al Ain Medical District Human Research Ethics Committee.

## Abbreviations

AHS: Ambulatory health service
BCSP: Bowel Cancer Screening Program
BMI: Body mass index
EPI: Enterprise patient identifier
FIT: Fecal immunochemical test
gFOBT: Guaiac fecal occult blood testing
HAAD: Health Authority - Abu Dhabi
SKMC: Sheikh Khalifa Medical City
UAE: United Arab Emirates
UKBSP: UK Bowel screening program
USPSTF: United States of America preventive task force
